# Two-sample Mendelian randomization analysis of 91 circulating inflammatory protein levels and amyotrophic lateral sclerosis

**DOI:** 10.3389/fnagi.2024.1367106

**Published:** 2024-03-27

**Authors:** Chenxu Xiao, Xiaochu Gu, Yu Feng, Jing Shen

**Affiliations:** ^1^The Affiliated Jiangsu Shengze Hospital of Nanjing Medical University, Suzhou, China; ^2^Medical Laboratory, Suzhou Psychiatric Hospital, The Affiliated Guangji Hospital of Soochow University, Suzhou, China; ^3^The University of New South Wales, Kensington, NSW, Australia; ^4^The University of Melbourne, Parkville, VIC, Australia

**Keywords:** amyotrophic lateral sclerosis, circulating inflammatory protein, two-sample mendelian randomization, osteoprotegerin, tumor necrosis factor

## Abstract

**Introduction:**

Amyotrophic Lateral Sclerosis (ALS) is a neurodegenerative disease with poorly understood pathophysiology. Recent studies have highlighted systemic inflammation, especially the role of circulating inflammatory proteins, in ALS.

**Methods:**

This study investigates the potential causal link between these proteins and ALS. We employed a two-sample Mendelian Randomization(MR) approach, analyzing data from large-scale genome-wide association studies to explore the relationship between 91 circulating inflammatory proteins and ALS. This included various MR methods like MR Egger, weighted median, and inverse-variance weighted, complemented by sensitivity analyses for robust results.

**Results:**

Significant associations were observed between levels of inflammatory proteins, including Adenosine Deaminase, Interleukin-17C, Oncostatin-M, Leukemia Inhibitory Factor Receptor, and Osteoprotegerin, and ALS risk. Consistencies were noted across different *P*-value thresholds. Bidirectional MR suggested that ALS risk might influence levels of certain inflammatory proteins.

**Discussion:**

Our findings, via MR analysis, indicate a potential causal relationship between circulating inflammatory proteins and ALS. This sheds new light on ALS pathophysiology and suggests possible therapeutic targets. Further research is required to confirm these results and understand the specific roles of these proteins in ALS.

## 1 Introduction

Amyotrophic Lateral Sclerosis (ALS), also known as Lou Gehrig’s disease, is a neurodegenerative condition that primarily affects motor neurons. This disease is characterized by the progressive degeneration of motor neurons, leading to fatal paralysis. ALS manifests in two forms: familial and sporadic, differentiated by family history. Despite significant research efforts, the underlying pathogenesis of ALS remains elusive, and effective treatments are scarce (Ralli et al., 2019; Masrori and Van Damme, 2020; Yang et al., 2021).

In the last decade, substantial progress has been made in understanding the genetic architecture, pathophysiological mechanisms, and potential biomarkers of ALS (Yang et al., 2021; Witzel et al., 2022; Mead et al., 2023). These advancements have opened new avenues for therapeutic intervention, particularly in the domain of neuroinflammation, which is increasingly associated with ALS. Recent focus in ALS research has centered on systemic inflammation, especially the role of circulating inflammatory proteins (Henkel et al., 2004; Murdock et al., 2015; Zondler et al., 2016). Studies have linked proteins like PIKFYVE kinase and TDP-43 to neuronal damage in ALS, associating their inhibition or misfolding with neuronal injury (Gleixner et al., 2022; Shao et al., 2022; Tejwani et al., 2023). This growing body of evidence underscores the importance of these proteins as both biomarkers and potential therapeutic targets (Akiyama et al., 2022; Jiang et al., 2022; Hosaka et al., 2023).

Concurrently, the application of bioinformatics, molecular biology, and genetics in ALS research has significantly enhanced our understanding of the disease’s complexity (Sankaran et al., 2021; Carroll, 2022; Sari et al., 2022). Innovations in these fields have led to the identification of new genetic and molecular pathways in ALS, laying the groundwork for targeted therapies (Lehrach et al., 2011; Akinduro et al., 2021; Wu and Lin, 2022). One key methodology employed in this context is Mendelian Randomization (MR). MR leverages genetic variations as instrumental variables to establish causal relationships between exposures (inflammatory proteins) and outcomes (ALS), thus enhancing the credibility of causal inferences drawn from observational studies by reducing common confounders and reverse causation (Song et al., 2022; Lee et al., 2023; Liu et al., 2023; Rajasundaram and Gill, 2023).

Our study employs a two-sample MR approach, utilizing multiple *p*-value thresholds to increase the accuracy of our findings while acknowledging the trade-offs involved. Lower *p*-values, such as <5.0E−08, are typically used to ensure robustness of associations, reduce heterogeneity, and improve study precision. However, such stringent criteria may also exclude potentially meaningful associations. Therefore, by adopting different *p*-value thresholds, our analysis aims to strike a balance between minimizing false positives and not overlooking significant associations that could be crucial for understanding the pathophysiology of ALS (Liu et al., 2023; Soremekun et al., 2023).

## 2 Methods

**Figure 1:**
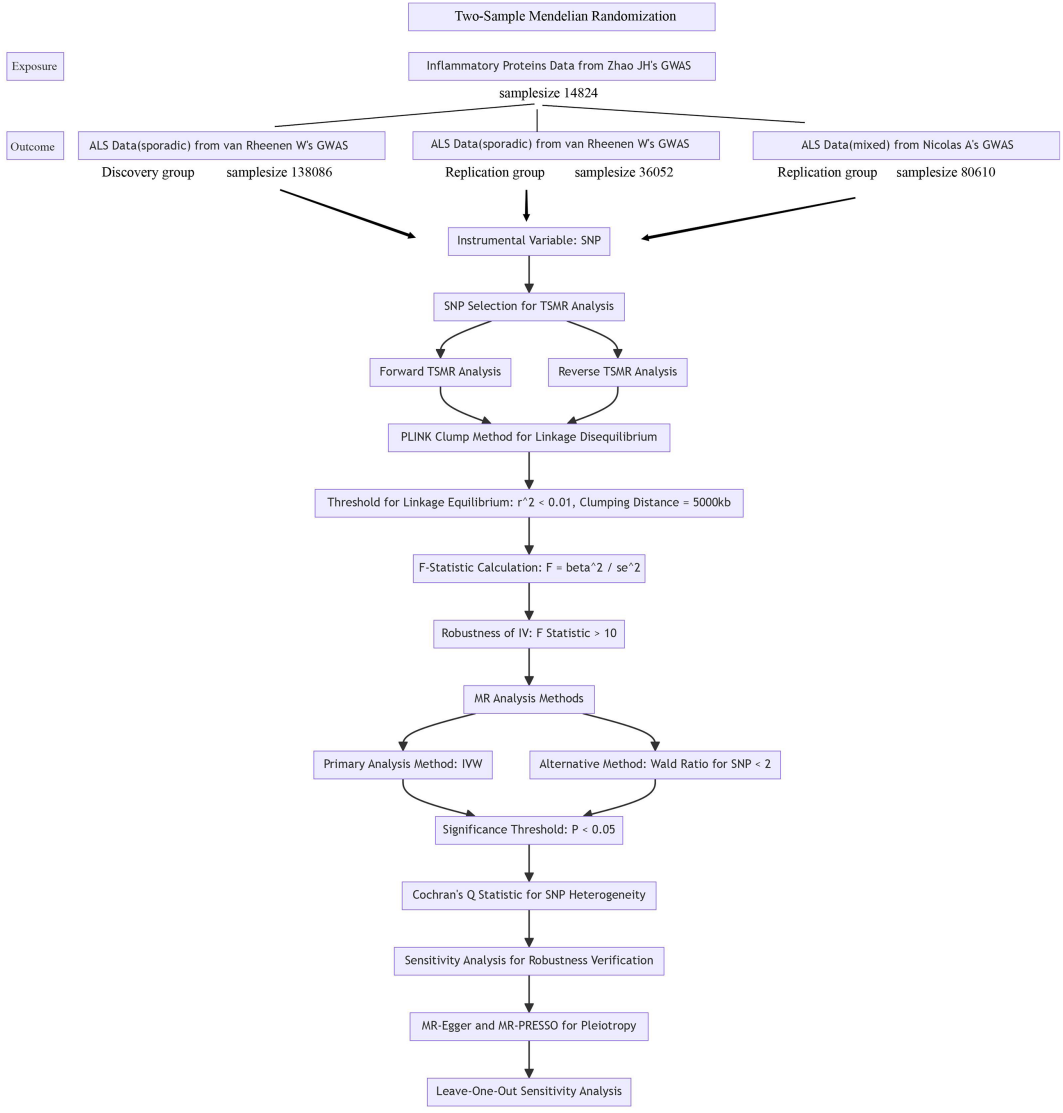
Flowchart figure.

The research process is illustrated in the flowchart figure (Figure 1).

### 2.1 Data sources

Data on 91 circulating inflammatory proteins were sourced from GWAS data measured using the Olink Target Inflammation panel across 11 cohorts, involving a total of 14,824 European ancestry participants (Zhao et al., 2023). Data on Amyotrophic Lateral Sclerosis were derived from a meta-analysis of GWAS by van Rheenen et al., “GCST90027164”,^1^ including 27,205 ALS cases and 110,881 European ancestry controls (van Rheenen et al., 2021) (Supplementary Table S1). The ALS validation group comprises two GWAS datasets curated by van Rheenen and Nicolas. The initial dataset consists of sporadic ALS cases, while the validation sets include one sporadic ALS dataset and another dataset that does not differentiate between familial or sporadic ALS (Supplementary Table S1). Specific details regarding data curation are thoroughly explained in the original articles.

### 2.2 Mendelian randomization

We utilized a two-sample MR (TSMR) analysis to explore the causal relationship between circulating inflammatory proteins and ALS. In our MR analysis, inflammatory proteins were the exposure of interest, ALS was the outcome, and SNPs were used as instrumental variables. The TSMR approach was based on the following assumptions: (I) instrumental variables are closely associated with the risk of inflammatory proteins; (II) instrumental variables affect ALS risk only through their impact on inflammatory proteins; (III) instrumental variables are independent of confounding factors (Davey Smith and Hemani, 2014).

We selected SNPs associated with inflammatory proteins across the whole genome (*p* < 5 × 10^−8^), (*p* < 5 × 10^−7^), and (*p* < 5 × 10^−6^) for forward TSMR analysis. For reverse TSMR analysis of ALS GWAS instrumental variables, we used SNPs (*p* < 5 × 10^−8^). Additionally, PLINK clumping was employed to calculate linkage disequilibrium between each exposure’s SNPs on the basis of the 1,000 Genomes European panel, using an *r*^2^ < 0.01 (clumping distance = 5,000 kb) as the threshold for SNPs in linkage equilibrium. The *F*-statistic was calculated using *F* = beta^2^/se^2^, with all *F*-statistics >10, indicating robustness of the instrumental variables.

Several MR methods were used, including MR Egger, weighted median, IVW, Wald ratio, simple mode, and weighted mode. IVW was selected as the primary analysis method, using Wald ratio when snp < 2, with a *p*-value <0.05 considered significant (Burgess et al., 2013; Verbanck et al., 2018). Cochran’s *Q* statistic was used to assess heterogeneity between individual SNPs. If no significant heterogeneity was observed (*p* < 0.05), a fixed-effect model was adopted (Hemani et al., 2018); otherwise, the causal significance relationship needed cautious interpretation. Sensitivity analyses were also conducted to verify the robustness of our results. Furthermore, MR-Egger and MR-PRESSO methods were employed to determine the presence of pleiotropy. The intercept obtained from MR-Egger regression was used to measure directional pleiotropy, and MR-PRESSO was used for enhanced detection of pleiotropy (Bowden et al., 2016). Steiger testing was performed to determine the direction of causality. Leave-one-out sensitivity analysis was conducted to determine whether individual SNPs had a significant impact on MR results.

### 2.3 Statistical software

All statistical analyses were performed using R software version 4.3.0.^2^ MR analysis and Steiger filtering were performed using the “TwoSampleMR” R package (Smith and Ebrahim, 2003; Emdin et al., 2017; The Telomeres Mendelian Randomization Collaboration et al., 2017). MR-PRESSO was carried out using the “MRPRESSO” R package.

### 2.4 Selection of external datasets for validation

During the initial exploration phase of the study, we refrained from using *p*-value correction to capture more potential associations. To ensure the reliability of our preliminary results, we chose to validate them using two external datasets. One dataset focuses on sporadic ALS, while the other does not distinguish between familial or sporadic ALS. By including both sporadic ALS and non-differentiated familial or sporadic ALS datasets, we can comprehensively assess the generalizability of our findings. In the preliminary screening process, we incorporated potentially key proteins previously identified, including 12 inflammation-related proteins. By utilizing these datasets, we are able to validate the associations observed in our study (van Rheenen et al., 2016; Nicolas et al., 2018).

## 3 Results

### 3.1 Forward Mendelian randomization results

**Figure 2:**
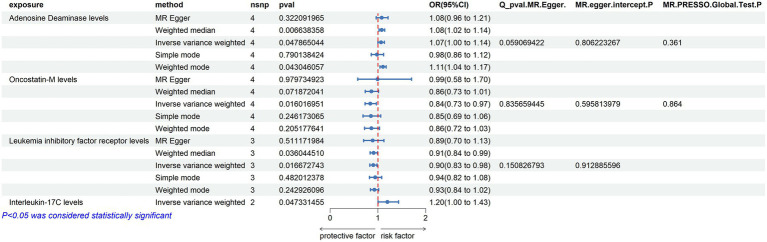
Adopt a significance threshold for selecting SNPs of *p* < 5 × 10^−8^. In this Mendelian randomization analysis, inflammatory proteins are analyzed as the exposure factor, with ALS as the resultant outcome. Significant findings are denoted by a P_IVW value less than 0.05.

Using the threshold of SNPs (*p* < 5 × 10^−8^), instrumental variables were extracted for 73 inflammatory proteins for TSMR analysis. The results indicated that increased levels of Adenosine Deaminase are associated with a higher risk of ALS (OR = 1.068, PIVW = 0.048). This analysis showed no significant heterogeneity (MR Egger *Q* = 5.658, *Q p*-value = 0.059) and no horizontal pleiotropy (*P* Egger Intercept = 0.806, *P* MR Presso = 0.361). An increase in Interleukin-17C levels was also found to increase ALS risk (OR = 1.199, PIVW = 0.047) (SNPs <3). Higher Oncostatin-M levels were associated with a decreased risk of ALS (OR = 0.84, PIVW = 0.016), with the analysis showing no significant heterogeneity (MR Egger *Q* = 0.359, *Q p*-value = 0.836) and no horizontal pleiotropy (*P* Egger Intercept = 0.596, *P* MR Presso = 0.864). Increased levels of Leukemia inhibitory factor receptor were associated with a decreased risk of ALS (OR = 0.903, PIVW = 0.017), with no significant heterogeneity (MR Egger *Q* = 2.064, *Q p*-value = 0.151) or horizontal pleiotropy (*P* Egger Intercept = 0.913) observed (Figure 2) (Supplementary Tables S2, S3, S8).

Due to the initial selection criteria, some proteins did not yield SNPs, leading us to relax the conditions. We selected SNPs with a threshold of *p* < 5 × 10^−7^ as instrumental variables, and conducted TSMR analysis on 86 inflammatory proteins. The results revealed the following:

Adenosine Deaminase Levels: an increase in Adenosine Deaminase levels was associated with an increased risk of ALS (OR = 1.07, PIVW = 0.025). The analysis showed no significant heterogeneity (MR Egger *Q* = 6.654, *Q p*-value = 0.084) and no evidence of horizontal pleiotropy (*P* Egger Intercept = 0.820, *P* MR Presso = 0.339).

Interleukin-5 Levels: higher levels of Interleukin-5 were also linked to an increased risk of ALS (OR = 1.5, PIVW = 0.015), with fewer than three SNPs involved.

SIR2-like Protein 2 Levels: an elevation in SIR2-like protein 2 levels was correlated with an increased risk of ALS (OR = 1.24, PIVW = 0.024), again with fewer than three SNPs.

Neurturin Levels: increased levels of Neurturin were found to raise the risk of ALS (OR = 1.237, PIVW = 0.040), with fewer than three SNPs.

Leukemia Inhibitory Factor Receptor Levels: conversely, an increase in Leukemia inhibitory factor receptor levels was associated with a decreased risk of ALS (OR = 0.912, PIVW = 0.029). This analysis also showed no significant heterogeneity (MR Egger *Q* = 2.966, *Q p*-value = 0.227) and no horizontal pleiotropy (*P* Egger Intercept = 0.713, *P* MR Presso = 0.566).

**Figure 3:**
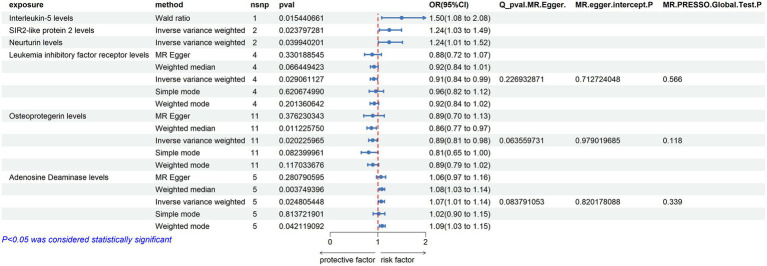
Adopt a significance threshold for selecting SNPs of *p* < 5 × 10^−7^. In this Mendelian randomization analysis, inflammatory proteins are analyzed as the exposure factor, with ALS as the resultant outcome. Significant findings are denoted by a P_IVW value less than 0.05.

Osteoprotegerin Levels: higher levels of Osteoprotegerin were linked to a reduced risk of ALS (OR = 0.89, PIVW = 0.020). The analysis indicated no significant heterogeneity (MR Egger *Q* = 16.163, *Q p*-value = 0.064) and no horizontal pleiotropy (*P* Egger Intercept = 0.979, *P* MR Presso = 0.118) (Figure 3) (Supplementary Tables S2, S4, S9).

In an effort to include more inflammatory proteins, the criteria were adjusted by setting the SNP threshold to *p* < 5 × 10^−6^. This enabled the extraction of instrumental variables for all 91 inflammatory proteins, which were then analyzed using TSMR. The findings were as follows:

ADA (Adenosine Deaminase) Levels: an increase in ADA levels was associated with an increased risk of ALS (OR = 1.072, PIVW = 0.037). This analysis indicated the presence of heterogeneity (MR Egger *Q* = 29.224, *Q p*-value = 0.004) and no evidence of horizontal pleiotropy (*P* Egger Intercept = 0.735, *P* MR Presso = 0.389).

TNF-beta Levels: elevated TNF-beta levels were associated with a reduced risk of ALS (OR = 0.951, PIVW = 0.012). The analysis showed heterogeneity (MR Egger *Q* = 34.518, *Q p*-value = 0.184) and no horizontal pleiotropy (*p* = 0.454, *P* MR Presso = 0.723).

Osteoprotegerin Levels: an increase in Osteoprotegerin levels was linked to a decreased risk of ALS (OR = 0.916, PIVW = 0.031). The analysis did not show significant heterogeneity (MR Egger *Q* = 27.915, *Q p*-value = 0.085) and no horizontal pleiotropy was found (*P* Egger Intercept = 0.440, *P* MR Presso = 0.125).

**Figure 4:**
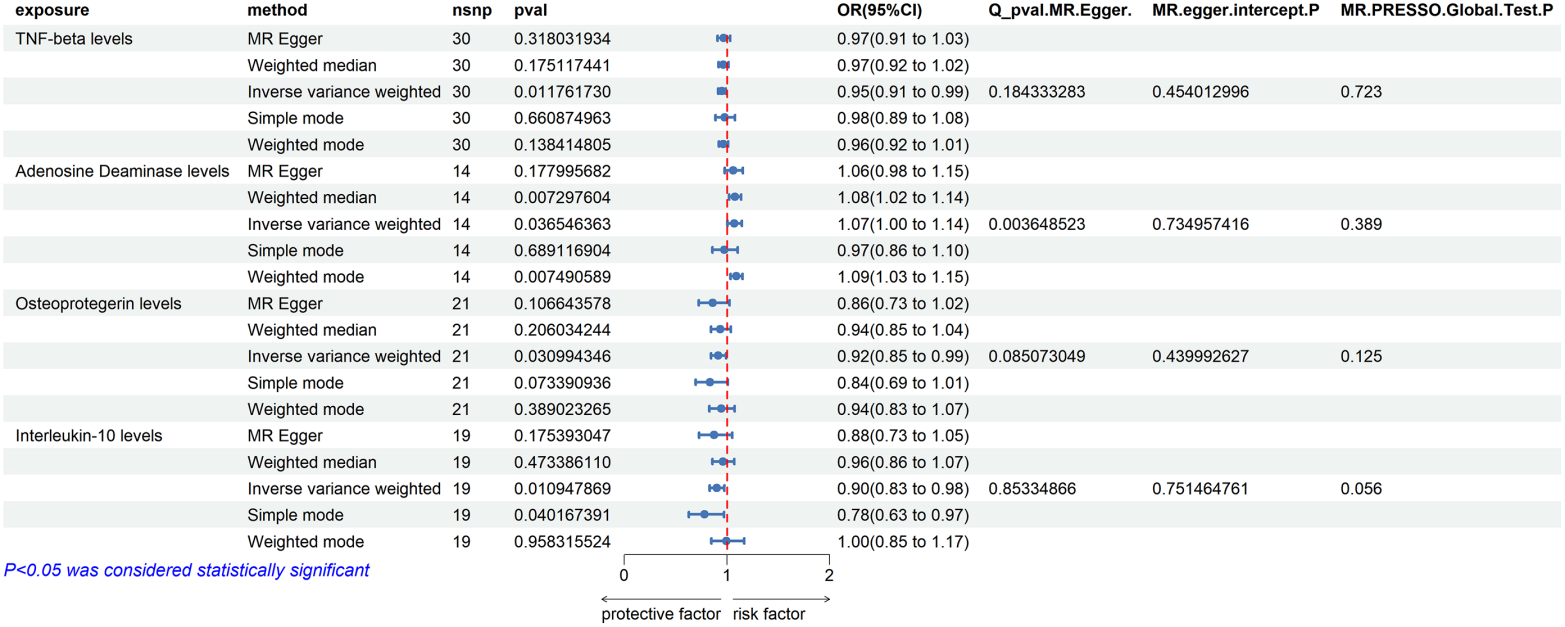
Adopt a significance threshold for selecting SNPs of *p* < 5 × 10^−6^. In this Mendelian randomization analysis, inflammatory proteins are analyzed as the exposure factor, with ALS as the resultant outcome. Significant findings are denoted by a P_IVW value less than 0.05.

Interleukin-10 Levels: higher levels of Interleukin-10 were associated with a decreased risk of ALS (OR = 0.901, PIVW = 0.011). This analysis indicated no significant heterogeneity (MR Egger *Q* = 11.062, *Q p*-value = 0.853) and no horizontal pleiotropy (*P* Egger Intercept = 0.751, *P* MR Presso = 0.056) (Figure 4) (Supplementary Tables S2, S5, S10).

From the three sets of analyses conducted, we can draw several conclusions:

ADA Levels: an increase in ADA levels was found to heighten the risk of ALS. This finding was significant across all three sets of analyses (*P* IVW < 0.05). Although there was heterogeneity in the results with the threshold at *p* < 5 × 10^−6^, the IVW results were relatively stable.

Leukemia Inhibitory Factor Receptor Levels: higher levels of the Leukemia inhibitory factor receptor were associated with a decreased risk of ALS. This was significantly observed in the first two sets of analyses (*P* IVW < 0.05).

Osteoprotegerin Levels: an increase in Osteoprotegerin levels also appeared to reduce the risk of ALS. This outcome was significant in the latter two sets of analyses (*P* IVW < 0.05).

**Figure 5:**
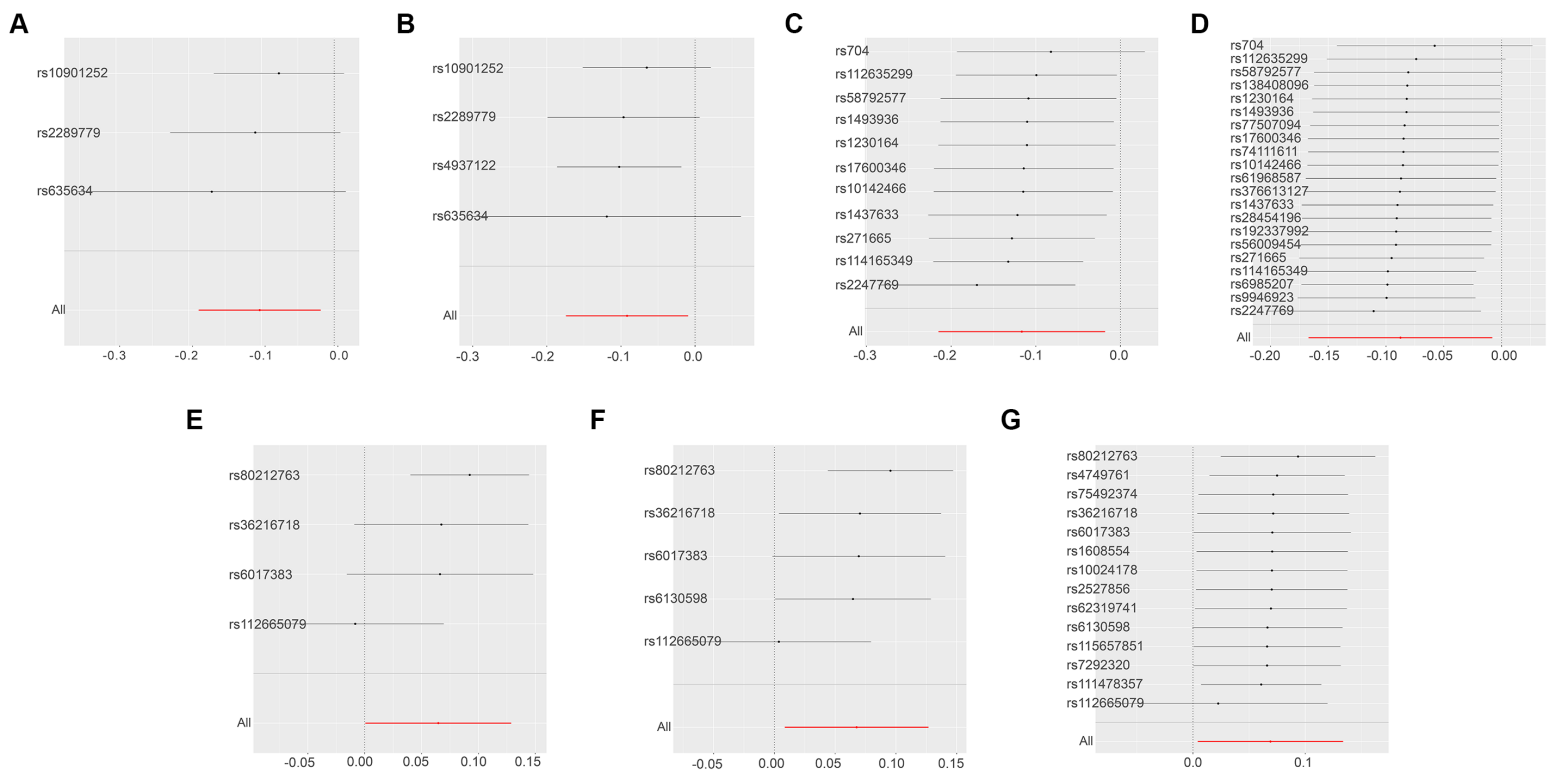
Displays the leave-one-out analysis results using the IVW method, assessing the impact of individual SNPs on the overall MR findings by sequentially excluding each SNP. The *Y*-axis corresponds to each excluded rsID and the aggregate IVW method result without any SNP exclusions. The *X*-axis represents the specific IVW values, where black and red dots denote beta effect values, and the lines indicate the confidence intervals of the beta values. Specifically, **(A,B)** illustrate the leukemia inhibitory factor receptor levels as the exposure, with SNP thresholds set at *p* < 5 × 10^−8^ and *p* < 5 × 10^−7^, respectively. **(C,D)** focus on osteoprotegerin levels as the exposure, applying SNP thresholds of *p* < 5 × 10^−7^ and *p* < 5 × 10^−6^. Finally, the levels of adenosine deaminase as the exposure factor are examined in **(E–G)**, with SNP thresholds set at *p* < 5 × 10^−8^, *p* < 5 × 10^−7^, and *p* < 5 × 10^−6^, respectively.

Next, we conducted a leave-one-out analysis on the three key results mentioned above. This involved sequentially excluding each SNP and estimating the effect sizes for the remaining SNPs. For both Leukemia inhibitory factor receptor levels and Osteoprotegerin levels, the analysis showed no significant difference in effect size before and after exclusion, indicating that no single SNP had a significant impact on the MR estimates. However, in the three sets of analyses for ADA levels, the exclusion of the SNP “rs112665079” led to a deviation in results, suggesting that rs112665079 has a significant influence on the MR estimation results (Figure 5) (Supplementary Table S7).

**Figure 6:**
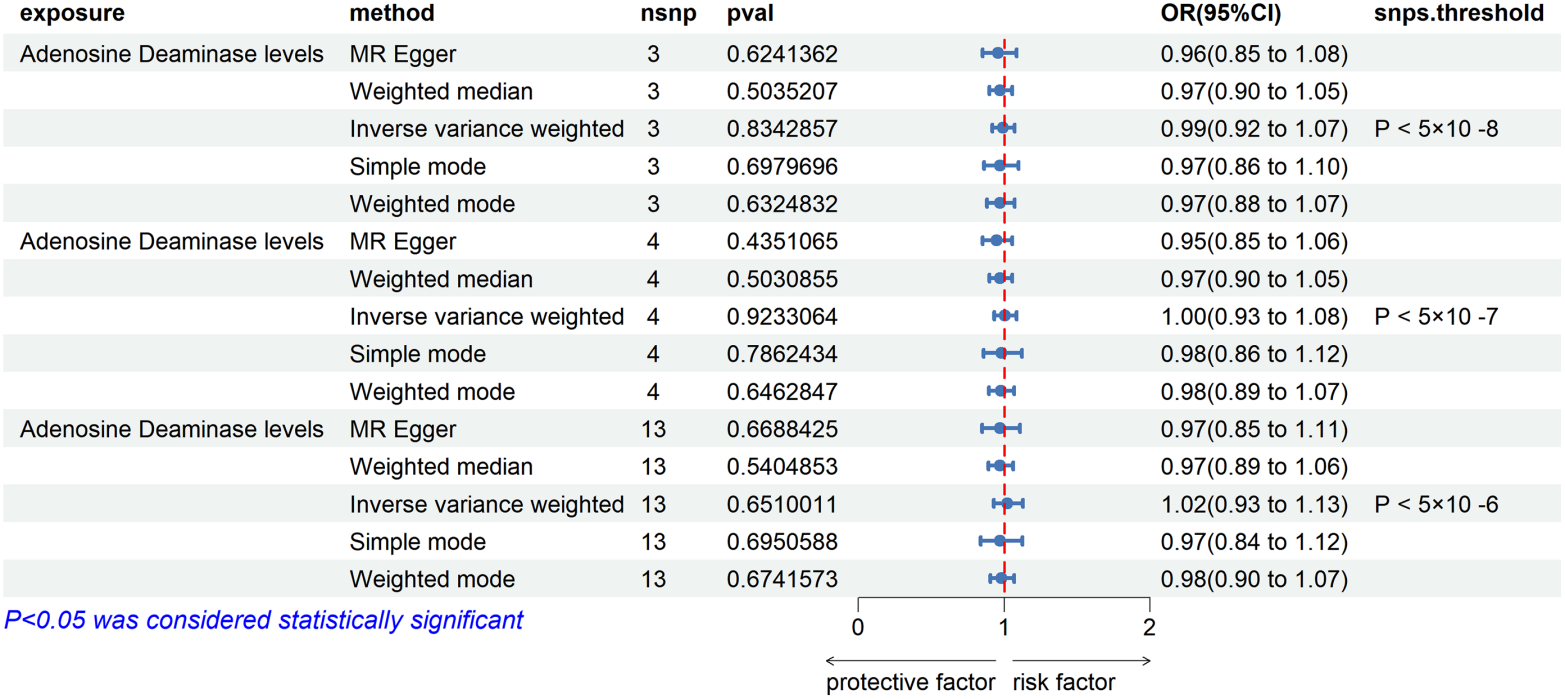
MR Results with Adenosine Deaminase Correction. SNP selection thresholds are applied with *p*-values <5 × 10^−8^, *p* < 5 × 10^−7^, and *p* < 5 × 10^−6^, respectively. Adenosine Deaminase protein is considered as the exposure factor, and ALS as the outcome in the Mendelian randomization analysis.

After excluding rs112665079 and reanalyzing TSMR with ADA levels as the exposure and ALS as the outcome, the results were contrary to the previous findings, showing no significant correlation. This indeed demonstrates the significant impact of rs112665079 on the MR estimation results (Figure 6).

### 3.2 Reverse Mendelian randomization results

In the reverse Mendelian randomization analysis involving ALS and 91 inflammatory proteins, the following results were obtained:

C-C Motif Chemokine 20 Levels: an increase in the risk of ALS was associated with elevated levels of C-C motif chemokine 20 (OR = 1.089, PIVW = 0.020). The analysis showed no significant heterogeneity (MR Egger *Q* = 9.884, *Q p*-value = 0.626) and no horizontal pleiotropy (*P* Egger Intercept = 0.742, *P* MR Presso = 0.725).

Tumor Necrosis Factor Ligand Superfamily Member 12 Levels: similarly, an increased risk of ALS was associated with higher levels of Tumor necrosis factor ligand superfamily member 12 (OR = 1.097, PIVW = 0.010). No significant heterogeneity was observed in this analysis (MR Egger *Q* = 7.787, *Q p*-value = 0.802), and there was no evidence of horizontal pleiotropy (*P* Egger Intercept = 0.127, *P* MR Presso = 0.586).

**Figure 7:**
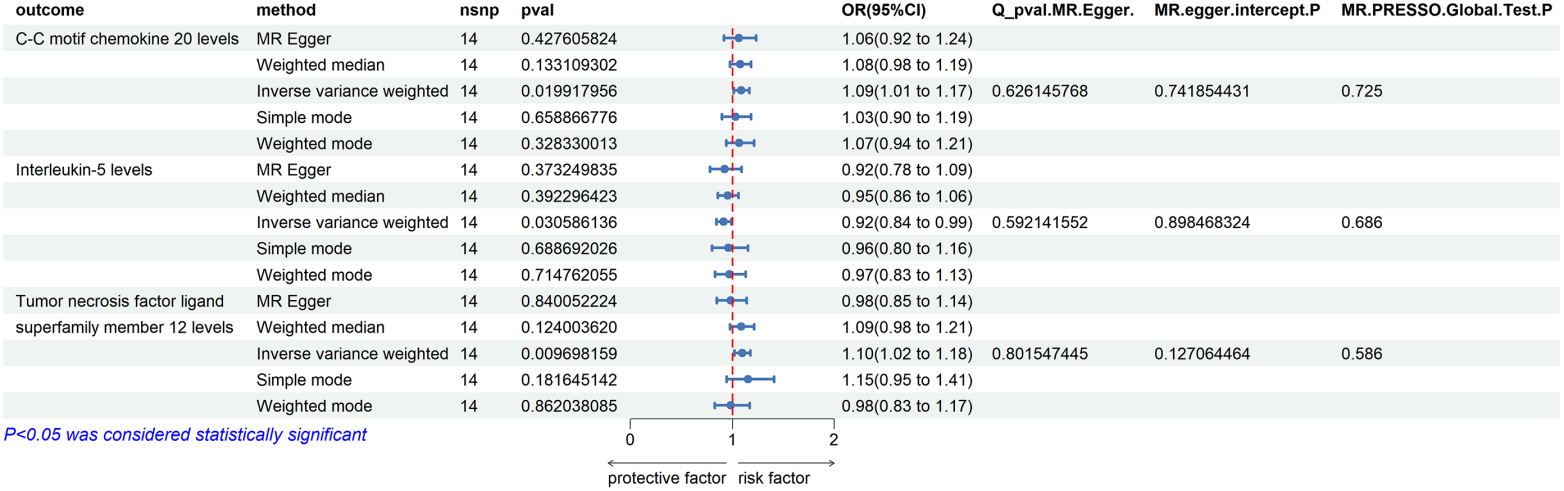
Reverse MR Results. Mendelian randomization analysis with ALS as the exposure factor and inflammatory proteins as the outcome, considering significance with p_ivw < 0.05.

Interleukin-5 Levels: in contrast, an increased risk of ALS was associated with decreased levels of Interleukin-5 (OR = 0.915, PIVW = 0.031). This analysis also showed no significant heterogeneity (MR Egger *Q* = 10.272, *Q p*-value = 0.592) and no horizontal pleiotropy (*P* Egger Intercept = 0.898, *P* MR Presso = 0.686) (Figure 7).

**Figure 8:**
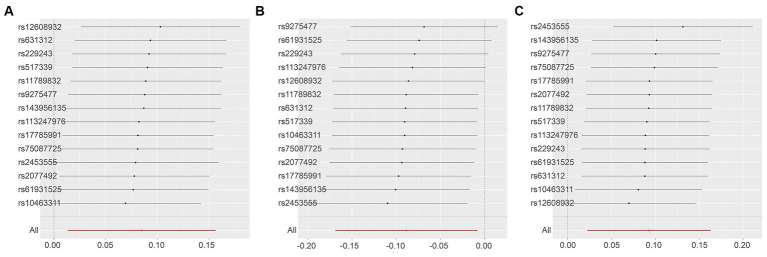
Leave-One-Out Analysis Results for Various Outcomes with ALS Exposure. This series illustrates the influence of ALS as an exposure factor on different inflammatory markers, using the leave-one-out methodology to assess the impact of individual SNPs on the overall results. **(A)**: C-C motif chemokine 20 levels as the outcome, with ALS as the exposure; **(B)**: Interleukin-5 levels as the outcome, with ALS as the exposure; **(C)**: Tumor necrosis factor ligand superfamily member 12 levels as the outcome, with ALS as the exposure.

A sensitivity analysis using the leave-one-out approach demonstrated robust results (Figure 8) (Supplementary Tables S2, S6, S11).

### 3.3 Validation group results

**Figure 9:**
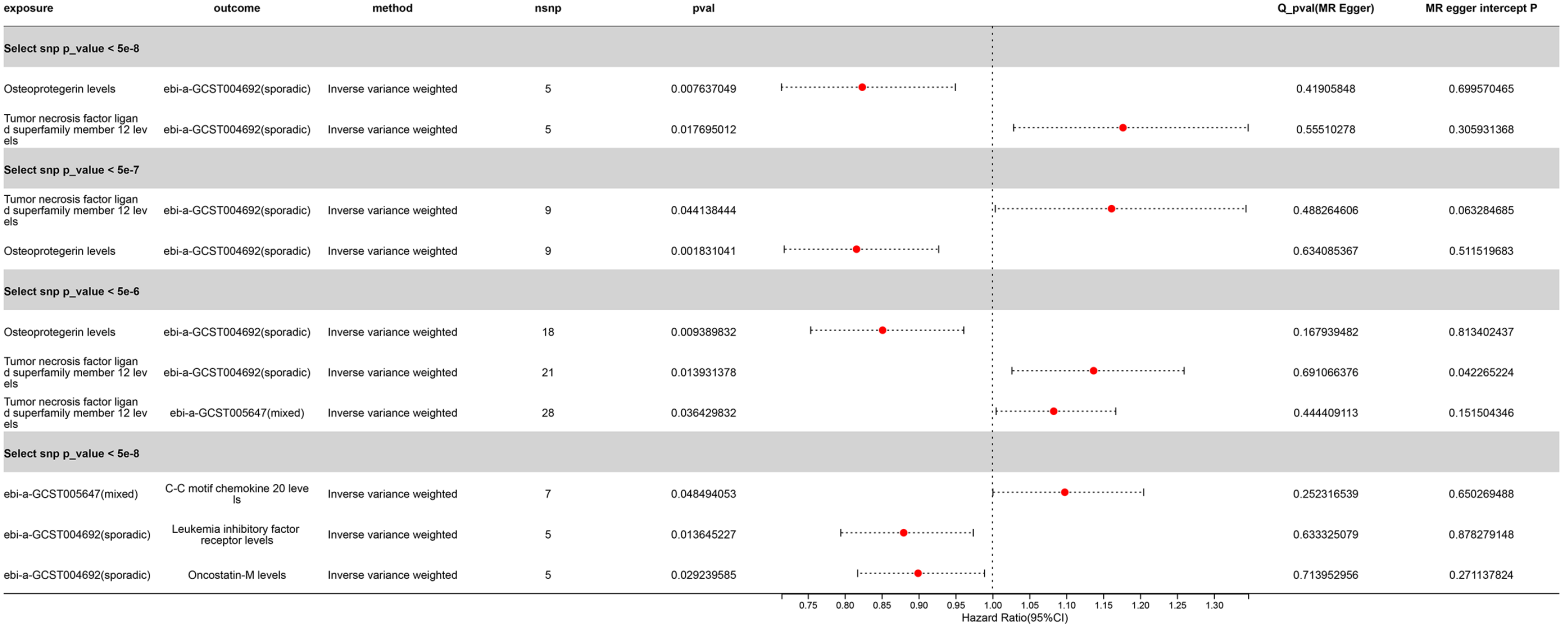
Significant Mendelian Randomization Results of Inflammatory Proteins with ALS Validation Sets (p_ivw < 0.05 denotes significance). “Sporadic” denotes a cohort of sporadic ALS, while “Mixed” refers to a combined cohort of familial and sporadic ALS.

We selected three SNP thresholding tool variables (*p* < 5 × 10^−8^, *p* < 5 × 10^−7^, *p* < 5 × 10^−6^) and 12 inflammatory proteins as exposures, with ALS as the outcome, for two-sample Mendelian randomization analysis. The clumping conditions are the same as those for the test set. We were surprised to find that, whether in the initial exploration phase or in the validation set, the levels of osteoprotegerin showed significance for sporadic ALS (OR < 1, PIVW < 0.05) (Figure 9) (Supplementary Tables S2–S4). This result suggests that there may be an association between Osteoprotegerin levels and sporadic ALS, where Osteoprotegerin levels may play a protective role.

Another remarkable discovery is the correlation of Tumor Necrosis Factor Ligand Superfamily Member 12 levels with the sporadic ALS validation set across three different SNP threshold groups, with a potential association observed with the mixed ALS set at SNP (*p* < 5 × 10^−6^) (OR > 1, *P* IVW < 0.05) (Figure 9) (Supplementary Tables S2–S4). This suggests that an increase in Tumor necrosis factor ligand superfamily member 12 levels may be a risk factor for ALS. However, we observed the opposite causal relationship in the initial GWAS set.

These results suggest complex interactions between ALS and Tumor Necrosis Factor Ligand Superfamily Member 12 levels, possibly involving bidirectional causal relationships.

## 4 Discussion

This study utilized the TSMR approach to explore the potential causal relationship between circulating inflammatory proteins and ALS. Our analysis revealed several significant associations between inflammatory proteins and the risk of ALS, offering new insights into the pathophysiology of ALS and potentially unveiling new therapeutic targets.

In our study, different *p*-value thresholds significantly impacted the results. Lower *p*-value thresholds (e.g., < 5.0E−08) are commonly employed to ensure robustness of associations, reduce heterogeneity, and enhance the accuracy of the research. However, such stringent criteria might also exclude potentially meaningful associations (Bottigliengo et al., 2022; Chen et al., 2023; Liu et al., 2023; Ren et al., 2023). Our analysis indicated that some associations, previously insignificant, became significant when the *p*-value threshold was relaxed, underscoring the importance and complexity of *p*-value selection in research on the relationship between inflammatory proteins and ALS.

Our study employed both forward and reverse MR analyses, a method that allows for a more comprehensive exploration of the potential causal relationship between inflammatory proteins and ALS (Perry et al., 2021; Huimei Huang et al., 2022; Wang et al., 2022; Yin et al., 2023). The forward MR analysis revealed associations between increased levels of specific inflammatory proteins and an increased risk of ALS, whereas the reverse MR analysis provided evidence that an increased risk of ALS could lead to changes in certain inflammatory protein levels. These findings suggest a possible bidirectional causal relationship between inflammatory proteins and ALS, further complicating their interaction.

In our research, multiple inflammatory proteins identified across various *p*-value thresholds showed significant positive results related to ALS risk. For instance, increased levels of ADA were significantly associated with an increased risk of ALS. Previous studies have suggested that ADA may play an important role in neurodegenerative diseases, linked to neuronal damage and inflammatory responses. Allen et al. identified a defect in adenosine to inosine deamination in astrocytes of ALS patients caused by reduced ADA expression. This defect led to increased sensitivity to adenosine-mediated toxicity (Allen et al., 2019). Supplementing inosine could reverse motor neuron toxicity observed in co-cultured patient astrocytes (Allen et al., 2018). Song et al. explored gene therapy for ALS by upregulating ADAR2 in mouse motor neurons using adeno-associated viral vectors. This treatment prevented progressive motor dysfunction and rescued motor neurons from death by normalizing TDP-43 expression, suggesting a potential gene therapy approach for ALS (Song and Pan, 2014).

In summary, this section of the study discusses the significant associations found between changes in Leukemia inhibitory factor receptor and Osteoprotegerin levels and the risk of ALS. It highlights the diverse roles of cytokines, including LIFR, in skeletal muscle physiology and their impact on muscle cell growth, differentiation, metabolism, nerve innervation, and inflammatory cell recruitment to muscle injury sites (Hunt and White, 2016). The research also touches on the limited but emerging findings linking AIFR and Osteoprotegerin to ALS, as well as their roles in neuropsychiatric disorders, emphasizing the importance of inflammation and immune mechanisms in these conditions (Ham et al., 2018; Hashioka et al., 2019; Novellino et al., 2020; Xu et al., 2023).

Furthermore, the study finds significant correlations between ALS risk and changes in levels of various inflammatory proteins, such as Interleukin-17C, Oncostatin-M, Interleukin-5 levels, SIR2-like protein 2 levels, Neurturin levels, TNF-beta levels and Interleukin-10, under different *p*-value thresholds. Reverse MR analysis suggests that increased ALS risk could lead to changes in certain inflammatory protein levels, such as motif chemokine 20 levels, Tumor necrosis factor ligand superfamily member 12 levels and Interleukin-5 levels. These findings offer new perspectives for research into the roles of these proteins in neuroprotection, neuroregeneration, and inflammation, potentially contributing to understanding and treating ALS and related neuropsychiatric disorders.

In summary, our study, based on GWAS data from European populations, suggests that Osteoprotegerin levels confer a protective effect against sporadic ALS, validated in two datasets. Additionally, we observed a complex bidirectional relationship between Tumor Necrosis Factor Ligand Superfamily Member 12 levels and sporadic ALS. Furthermore, some correlations were found in the GWAS dataset combining Tumor Necrosis Factor Ligand Superfamily Member 12 with familial and sporadic ALS, highlighting the potential complex bidirectional association between Tumor Necrosis Factor Ligand Superfamily Member 12 levels and ALS. Future research can delve into the specific roles of Osteoprotegerin and Tumor Necrosis Factor Ligand Superfamily Member 12 in the pathogenesis of ALS, assess their potential as biomarkers, and explore therapeutic strategies targeting them.

The findings of this study rely on data from the European population, implying that the applicability of its conclusions may have certain limitations. Although these inflammatory proteins show significant associations for some ALS patients within the European population, we must acknowledge that ALS patients in other populations worldwide may exhibit different levels of correlation and significance. Therefore, to comprehensively understand the role of these inflammatory proteins and their differences across diverse populations, future research should focus on collecting and analyzing data from more varied population groups. Such research endeavors will help uncover the population-specific aspects of ALS pathogenesis, thereby laying the groundwork for the discovery of universally applicable therapeutic strategies.

## 5 Conclusion

In summary, our study offers new insights into the role of circulating inflammatory proteins in ALS and paves the way for future research and the development of therapeutic strategies. Future research should focus on validating these findings and exploring the relationships between other potential inflammatory proteins and ALS. Furthermore, a deeper investigation into the specific roles of these inflammatory proteins in ALS pathophysiology will be crucial.

## Data availability statement

The original contributions presented in the study are included in the article/Supplementary material, further inquiries can be directed to the corresponding author.

## Author contributions

CX: Conceptualization, Data curation, Formal analysis, Funding acquisition, Investigation, Methodology, Project administration, Resources, Software, Supervision, Validation, Visualization, Writing – original draft, Writing – review & editing. XG: Writing – original draft, Writing – review & editing. YF: Writing – original draft, Writing – review & editing. JS: Conceptualization, Data curation, Formal analysis, Funding acquisition, Investigation, Methodology, Project administration, Resources, Software, Supervision, Validation, Visualization, Writing – original draft, Writing – review & editing.

## Footnotes

^1^ https://www.ebi.ac.uk/gwas/studies/GCST90027164

^2^ https://www.r-project.org/

## References

[ref1] Akinduro O. O. Suarez-Meade P. Garcia D. Brown D. A. Sarabia-Estrada R. Attia S.. (2021). Targeted therapy for Chordoma: key molecular Signaling pathways and the role of multimodal therapy. Target. Oncol. 16, 325–337. doi: 10.1007/s11523-021-00814-5, PMID: 33893940 PMC9286495

[ref2] Akiyama T. Koike Y. Petrucelli L. Gitler A. (2022). Cracking the cryptic code in amyotrophic lateral sclerosis and frontotemporal dementia: towards therapeutic targets and biomarkers. Clin. Transl. Med. 12:e818. doi: 10.1002/ctm2.818, PMID: 35567447 PMC9098226

[ref3] Allen S. P. Hall B. Castelli L. Francis L. Woof R. Higginbottom A.. (2018). Inosine reverses motor neuron toxicity observed in amyotrophic lateral sclerosis patient astrocytes with an adenosine deaminase deficiency. Biochimica et Biophysica Acta 1859:e23. doi: 10.1016/J.BBABIO.2018.09.071

[ref4] Allen S. P. Hall B. Castelli L. Francis L. Woof R. Siskos A.. (2019). Astrocyte adenosine deaminase loss increases motor neuron toxicity in amyotrophic lateral sclerosis. Brain 142:586. doi: 10.1093/brain/awy353, PMID: 30698736 PMC6391613

[ref5] Bottigliengo D. Foco L. Seibler P. Klein C. König I. Del Greco M. (2022). A Mendelian randomization study investigating the causal role of inflammation on Parkinson’s disease. Brain 145, 3444–3453. doi: 10.1093/brain/awac193, PMID: 35656776 PMC9586538

[ref6] Bowden J. Davey Smith G. Haycock P. C. Burgess S. (2016). Consistent estimation in Mendelian randomization with some invalid instruments using a weighted median estimator. Genet. Epidemiol. 40, 304–314. doi: 10.1002/gepi.21965, PMID: 27061298 PMC4849733

[ref7] Burgess S. Butterworth A. Thompson S. G. (2013). Mendelian randomization analysis with multiple genetic variants using summarized data. Genet. Epidemiol. 37, 658–665. doi: 10.1002/gepi.21758, PMID: 24114802 PMC4377079

[ref8] Carroll D. (2022). Integrating experience with databases, bioinformatics, and wet lab exercises for students in an introductory genetics course. Biochem. Mol. Biol. Educ. 50, 457–459. doi: 10.1002/bmb.21649, PMID: 35904089 PMC10661174

[ref9] Chen J. Song B. Feng Q. Feng Y. Yu Q. He P.. (2023). AB0154 MENDELIAN randomization study implies causal linkage between POLYMYOSITIS and physical ACTIVITY. Ann. Rheum. Dis. 82, 1256.2–1256.1256. doi: 10.1136/annrheumdis-2023-eular.4215

[ref10] Davey Smith G. Hemani G. (2014). Mendelian randomization: genetic anchors for causal inference in epidemiological studies. Hum. Mol. Genet. 23, R89–R98. doi: 10.1093/hmg/ddu328, PMID: 25064373 PMC4170722

[ref11] Emdin C. A. Khera A. V. Kathiresan S. (2017). Mendelian Randomization. JAMA 318, 1925–1926. doi: 10.1001/jama.2017.17219 29164242

[ref12] Gleixner A. Morris Verdone B. Otte C. G. Anderson E. N. Ramesh N. Shapiro O. R.. (2022). NUP62 localizes to ALS/FTLD pathological assemblies and contributes to TDP-43 insolubility. Nat. Commun. 13, 3380–3317. doi: 10.1038/s41467-022-31098-6, PMID: 35697676 PMC9192689

[ref13] Ham S. Kim T. Hong H. Kim Y. S. Tang Y. Im H. I. (2018). Big data analysis of genes associated with neuropsychiatric disorders in an Alzheimer’s disease animal model. Front. Neurosci. 12:407. doi: 10.3389/fnins.2018.00407, PMID: 29962931 PMC6013555

[ref14] Hashioka S. Inoue K. Miyaoka T. Hayashida M. Wake R. Oh-Nishi A.. (2019). The possible causal link of periodontitis to neuropsychiatric disorders: more than psychosocial mechanisms. Int. J. Mol. Sci. 20:3723. doi: 10.3390/ijms20153723, PMID: 31366073 PMC6695849

[ref15] Hemani G. Bowden J. Davey Smith G. (2018). Evaluating the potential role of pleiotropy in Mendelian randomization studies. Hum. Mol. Genet. 27, R195–r208. doi: 10.1093/hmg/ddy163, PMID: 29771313 PMC6061876

[ref16] Henkel J. S. Engelhardt J. I. Siklós L. Simpson E. P. Kim S. H. Pan T.. (2004). Presence of dendritic cells, MCP-1, and activated microglia/macrophages in amyotrophic lateral sclerosis spinal cord tissue. Ann. Neurol. 55, 221–235. doi: 10.1002/ana.10805, PMID: 14755726

[ref17] Hosaka T. Tsuji H. Kwak S. (2023). Roles of aging, circular RNAs, and RNA editing in the pathogenesis of amyotrophic lateral sclerosis: potential biomarkers and therapeutic targets. Cells 12:1443. doi: 10.3390/cells12101443, PMID: 37408276 PMC10216766

[ref18] Huimei Huang S. Cheng C.’e. L. Cheng B. Liu L. Xuena Yang P. Meng Y. Y.. (2022). Dissecting the association between psychiatric disorders and neurological proteins: a genetic correlation and two-sample bidirectional Mendelian randomization study. Neuropsychopharmacology 34, 311–317. doi: 10.1017/neu.2022.10, PMID: 35343424

[ref19] Hunt L. White J. (2016). The role of Leukemia inhibitory factor receptor Signaling in skeletal muscle growth, injury and disease. Advances in experimental medicine and biology, 900, 45–59. Springer, Cham 27003396 10.1007/978-3-319-27511-6_3

[ref20] Jiang Z. Z. Wang Z. Wei X. J. Yu X. F. (2022). Inflammatory checkpoints in amyotrophic lateral sclerosis: from biomarkers to therapeutic targets. Front. Immunol. 13:59994. doi: 10.3389/fimmu.2022.1059994, PMID: 36618399 PMC9815501

[ref21] Lee G. Yao C. Hwang S.-J. Ma J. Joehanes R. Lee D. H.. (2023). Integrative Mendelian randomization reveals the soluble receptor for advanced glycation end products as protective in relation to rheumatoid arthritis. Sci. Rep. 13:8002. doi: 10.1038/s41598-023-35098-4, PMID: 37198231 PMC10192300

[ref22] Lehrach H. Schäfer R. Schlag P. M. (2011). “Deep sequencing”und prädiktive Modellierung als Konzept therapeutischer Entscheidungsfindungen in der Onkologie [deep sequencing and predictive modeling as a concept for therapeutic decision-making in oncology]. Onkologe 17, 477–486. doi: 10.1007/s00761-011-2025-9

[ref23] Liu R. Shi X. Feng J. Piao J. Yang Z. Zhao Y.. (2023). Ischemic stroke and cerebral microbleeds: a two-sample bidirectional Mendelian randomization study. Neurol Ther 12, 1299–1308. doi: 10.1007/s40120-023-00500-w, PMID: 37270442 PMC10310681

[ref24] Liu W.-S. Zhang Y.-R. Ge Y.-J. Wang H. Cheng W. Yu J. (2023). Inflammation and brain structure in Alzheimer’s disease and other neurodegenerative disorders: A Mendelian randomization study10.1007/s12035-023-03648-6 37736795

[ref25] Masrori P. Van Damme P. (2020). Amyotrophic lateral sclerosis: a clinical review. Eur. J. Neurol. 27, 1918–1929. doi: 10.1111/ene.14393, PMID: 32526057 PMC7540334

[ref26] Mead R. J. Shan N. Reiser J. Marshall F. Shaw P. J. (2023). Amyotrophic lateral sclerosis: a neurodegenerative disorder poised for successful therapeutic translation. Nat. Rev. Drug Discov. 22, 185–212. doi: 10.1038/s41573-022-00612-2, PMID: 36543887 PMC9768794

[ref27] Murdock B. J. Bender D. E. Segal B. M. Feldman E. L. (2015). The dual roles of immunity in ALS: injury overrides protection. Neurobiol. Dis. 77, 1–12. doi: 10.1016/j.nbd.2014.12.015, PMID: 25726748

[ref28] Nicolas A. Kenna K. P. Renton A. E. Ticozzi N. Faghri F. Chia R.. (2018). Genome-wide analyses identify KIF5A as a novel ALS gene. Neuron 97, 1268–1283.e6. doi: 10.1016/j.neuron.2018.02.027, PMID: 29566793 PMC5867896

[ref29] Novellino F. Sacca V. Donato A. Zaffino P. Spadea M. Vismara M.. (2020). Innate immunity: a common denominator between neurodegenerative and neuropsychiatric diseases. Int. J. Mol. Sci. 21:1115. doi: 10.3390/ijms21031115, PMID: 32046139 PMC7036760

[ref30] Perry B. Burgess S. Jones H. Zammit S. Upthegrove R. Mason A.. (2021). The potential shared role of inflammation in insulin resistance and schizophrenia: a bidirectional two-sample mendelian randomization study. PLoS Med. 18:e1003455. doi: 10.1371/journal.pmed.1003455, PMID: 33711016 PMC7954314

[ref31] Rajasundaram S. Gill D. (2023). Tumour necrosis factor receptor 1 inhibition and cardiovascular disease: A cis-Mendelian randomization study

[ref32] Ralli M. Lambiase A. Artico M. de Vincentiis M. Greco A. (2019). Amyotrophic lateral sclerosis: autoimmune pathogenic mechanisms, clinical features, and therapeutic perspectives. Israel Med. Assoc. J. 21, 438–443. 31507117

[ref33] Ren F. Jin Q.-Y. Qian Y. Ren X. Liu T. Zhan Y. (2023). Genetic evidence supporting the causal role of gut microbiota in chronic kidney disease and chronic systemic inflammation in CKD: a bilateral two-sample Mendelian randomization study. Front. Immunol. 14:87698. doi: 10.3389/fimmu.2023.1287698, PMID: 38022507 PMC10652796

[ref34] Sankaran S. M. Smith J. D. Roy K. R. (2021). CRISPR-Cas9 gene editing in yeast: a molecular biology and bioinformatics laboratory module for undergraduate and high school students. J. Microbiol. Biol. Educ. 22:e21. doi: 10.1128/jmbe.00106-21, PMID: 34594460 PMC8442027

[ref35] Sari I. J. Pongsophon P. Vongsangnak W. Pimthong P. Pitiporntapin S. (2022). The development of molecular genetics concept test for senior high school students using Rasch analysis. Int. J. Eval. Res. Educ. 11, 1687–1458. doi: 10.11591/ijere.v11i4.21846

[ref36] Shao W. Todd T. W. Wu Y. Jones C. Y. Tong J. Jansen-West K. R.. (2022). Two FTD-ALS genes converge on the endosomal pathway to induce TDP-43 pathology and degeneration. Science 378:94. doi: 10.1126/science.abq7860, PMID: 36201573 PMC9942492

[ref37] Smith G. D. Ebrahim S. (2003). Mendelian randomization: can genetic epidemiology contribute to understanding environmental determinants of disease? Int. J. Epidemiol. 32, 1–22. doi: 10.1093/ije/dyg070, PMID: 12689998

[ref38] Song C. J. Bian M. Y. Lei L. Chen L. L. (2022). Mendelian randomization and its application in periodontitis10.3760/cma.j.cn112144-20220228-00077 36266083

[ref39] Song Y. Pan W. (2014). Exploration of the pathogenesis of amyotrophic lateral sclerosis from the perspective of motor neuron TDP-43 protein expression and ADAR2 Activity. Neurodegener. Dis. 1, 119–125. doi: 10.1159/000368927

[ref40] Soremekun O. S. Musanabaganwa C. Uwineza A. Ardissino M. Rajasundaram S. Wani A.. (2023). Transl Psychiatry 13:2542. doi: 10.1038/s41398-023-02542-y, PMID: 37391434 PMC10313806

[ref41] Tejwani L. Jung Y. Kokubu H. Sowmithra S. Ni L. Lee C.. (2023). Reduction of nemo-like kinase increases lysosome biogenesis and ameliorates TDP-43–related neurodegeneration. J. Clin. Invest. 133:e138207. doi: 10.1172/JCI138207, PMID: 37384409 PMC10425213

[ref42] The Telomeres Mendelian Randomization Collaboration Haycock P. C. Burgess S. Nounu A. Zheng J. Okoli G. N.. (2017). Association between telomere length and risk of cancer and non-neoplastic diseases: a Mendelian randomization study. JAMA Oncol. 3, 636–651. doi: 10.1001/jamaoncol.2016.5945, PMID: 28241208 PMC5638008

[ref43] van Rheenen W. Shatunov A. Dekker A. M. McLaughlin R. L. Diekstra F. P. Pulit S. L.. (2016). Genome-wide association analyses identify new risk variants and the genetic architecture of amyotrophic lateral sclerosis. Nat. Genet. 48, 1043–1048. doi: 10.1038/ng.3622, PMID: 27455348 PMC5556360

[ref44] van Rheenen W. van der Spek R. A. A. Bakker M. K. van Vugt J. J. F. A. Hop P. J. Zwamborn R. A. J.. (2021). Common and rare variant association analyses in amyotrophic lateral sclerosis identify 15 risk loci with distinct genetic architectures and neuron-specific biology. Nat. Genet. 53, 1636–1648. doi: 10.1038/s41588-021-00973-1, PMID: 34873335 PMC8648564

[ref45] Verbanck M. Chen C. Y. Neale B. do R. (2018). Detection of widespread horizontal pleiotropy in causal relationships inferred from Mendelian randomization between complex traits and diseases. Nat. Genet. 50, 693–698. doi: 10.1038/s41588-018-0099-7, PMID: 29686387 PMC6083837

[ref46] Wang Q. Shi Q. Jiawen L. Wang Z. Hou J. (2022). Causal relationships between inflammatory factors and multiple myeloma: a bidirectional Mendelian randomization study. Int. J. Cancer 151, 1750–1759. doi: 10.1002/ijc.34214, PMID: 35841389

[ref47] Witzel S. Mayer K. Oeckl P. (2022). Biomarkers for amyotrophic lateral sclerosis. Curr. Opin. Neurol. 35, 699–704. doi: 10.1097/WCO.0000000000001094 35942674

[ref48] Wu J. Lin Z. (2022). Non-small cell lung cancer targeted therapy: drugs and mechanisms of drug resistance. Int. J. Mol. Sci. 23:15056. doi: 10.3390/ijms232315056, PMID: 36499382 PMC9738331

[ref49] Xu H. Zheng L. Wang L. Gao H. Wei Y. Chen J. (2023). Albumin and associated biomarkers in severe neuropsychiatric disorders: acute-phase schizophrenia and bipolar disorder. Neuropsychiatr. Dis. Treat. 19, 2027–2037. doi: 10.2147/NDT.S423399, PMID: 37790800 PMC10544194

[ref50] Yang X. Ji Y. Wang W. Zhang L. Chen Z. Yu M.. (2021). Amyotrophic lateral sclerosis: molecular mechanisms, biomarkers, and therapeutic strategies. Antioxidants (Basel) 10:1012. doi: 10.3390/antiox10071012, PMID: 34202494 PMC8300638

[ref51] Yin Z. Chen J. Xia M. Zhang X.-y. Li Y. Chen Z.-h.. (2023). Assessing causal relationship between circulating cytokines and age-related neurodegenerative diseases: a bidirectional two-sample Mendelian randomization analysis. Sci. Rep. 13:12325. doi: 10.1038/s41598-023-39520-9, PMID: 37516812 PMC10387057

[ref52] Zhao J. H. Stacey D. Eriksson N. Macdonald-Dunlop E. Hedman Å. K. Kalnapenkis A.. (2023). Genetics of circulating inflammatory proteins identifies drivers of immune-mediated disease risk and therapeutic targets. Nat. Immunol. 24, 1540–1551. doi: 10.1038/s41590-023-01588-w, PMID: 37563310 PMC10457199

[ref53] Zondler L. Müller K. Khalaji S. Bliederhäuser C. Ruf W. P. Grozdanov V.. (2016). Peripheral monocytes are functionally altered and invade the CNS in ALS patients. Acta Neuropathol. 132, 391–411. doi: 10.1007/s00401-016-1596-9, PMID: 26910103

